# The Roles of Cystatin B in the Brain and Pathophysiological Mechanisms of Progressive Myoclonic Epilepsy Type 1

**DOI:** 10.3390/cells13020170

**Published:** 2024-01-16

**Authors:** Shekhar Singh, Riikka H. Hämäläinen

**Affiliations:** A.I. Virtanen Institute for Molecular Sciences, University of Eastern Finland, Neulaniementie 2, 70211 Kuopio, Finland; shekhar.singh@uef.fi

**Keywords:** EPM1, Unverricht–Lundborg disease, Cystatin B, Stefin B, epilepsy, GABAergic neurons

## Abstract

Progressive myoclonic epilepsy type 1 (EPM1) is an autosomal recessive disorder, also known as Unverricht–Lundborg disease (ULD). EPM1 patients suffer from photo-sensitive seizures, stimulus-sensitive myoclonus, nocturnal myoclonic seizures, ataxia and dysarthria. In addition, cerebral ataxia and impaired GABAergic inhibition are typically present. EPM1 is caused by mutations in the Cystatin B gene (*CSTB*). The CSTB protein functions as an intracellular thiol protease inhibitor and inhibits Cathepsin function. It also plays a crucial role in brain development and regulates various functions in neurons beyond maintaining cellular proteostasis. These include controlling cell proliferation and differentiation, synaptic functions and protection against oxidative stress, likely through regulation of mitochondrial function. Depending on the differentiation stage and status of neurons, the protein localizes either to the cytoplasm, nucleus, lysosomes or mitochondria. Further, CSTB can also be secreted to the extracellular matrix for interneuron rearrangement and migration. In this review, we will review the various functions of CSTB in the brain and discuss the putative pathophysiological mechanism underlying EPM1.

## 1. Introduction

Progressive myoclonus epilepsies (PMEs) are a group of rare genetic symptomatic epilepsies, including Unverricht–Lundborg disease, Lafora disease, Batten disease and Neuronal ceroid lipofuscinoses (NCLs) [1]. PMEs are characterized by myoclonus, tonic–clonic seizures and progressive neurologic decline. The age of onset varies from infancy to adulthood, but most commonly, the first symptoms start in late childhood or during adolescence. The motor skills, balance and cognitive function of PME patients decline over time, and most patients present muscle rigidity, balance issues and unsteadiness in addition to seizures. The treatment of PMEs is mainly symptomatic for the seizures and myoclonus, accompanied by supportive actions, and the prognosis varies significantly between specific disorders [2].

Unverricht–Lundborg disease (ULD or progressive myoclonic epilepsy type 1, EPM1) is one of the most common forms of PMEs. It is an autosomal recessive disorder that leads to an onset of neurodegeneration between 6 and 15 years of age. The occurrence of EMP1 is higher in Finland than elsewhere in the world, with an estimated frequency of 1 in 20,000 in the Finnish population [3]. Initially, EPM1 was known as two separate diseases, the Baltic myoclonus and the Mediterranean myoclonus, depending on the origin of the patient. However, once the underlying mutations were identified in the *CSTB* gene, the two entities were recognized as the same disease, with the differences in prognosis between patients likely arising from varying treatment regimens [4].

**Figure 1:**
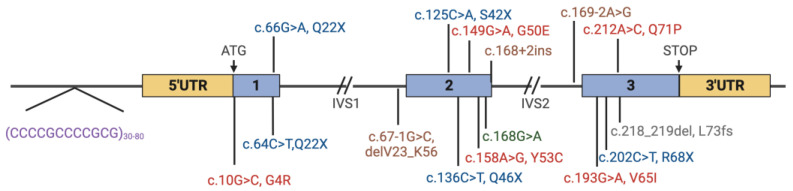
The structure of the *CSTB* gene and the locations of the reported mutations. Red = missense mutations, blue = nonsense mutations, green = silent mutations, brown = splice site mutations, grey = frameshift mutations, purple = promoter region expansion mutation. Created with Biorender.com (accessed on 5 December 2023).

EPM1 is caused by mutations in the Cystatin B (*CSTB*) gene, which encodes the CSTB protein that plays a crucial role in brain development. The CSTB protein is a protease inhibitor involved in the regulation of various cellular processes in neuronal cells, including cell cycle progression, cell proliferation and differentiation, apoptosis, transcriptional activity, mitochondrial function and synaptic function [5,6,7,8]. Moreover, it modulates neuronal distribution by attracting migrating interneurons [9,10]. To date, 19 different mutations have been reported in the *CSTB* gene in EPM1 patients (Figure 1, Table 1) that lead to neuronal dysfunction and neurodegeneration and the onset of the clinical disease [11,12,13].

**Table 1:** Published *CSTB* mutations identified in EPM1 patients.

Exon	gDNA (hg38)	cDNA	Protein (Reference)
1	g.43776260C>G	c.10G>C	p.Gly4Arg [14,15]
1	g.43776204C>T	c.66G>A	p.Gln22Ter [13]
intron 1	g.43774760C>G	c.67-1G>C	p.Gln22Ter [11,16,17,18]
2	g.43774701G>T	c.125C>A	p.Ser42Ter [19]
2	g.43774690G>A	c.136C>T	p.Gln46Ter [11]
2	g.43774677C>T	c.149G>A	p.Gly50Glu [20]
2	g.43774668T>C	c.158A>G	p.Try53Cys [21]
2	g.43774658C>T	c.168G>A	p.Lys56= [22]
2 intron	g.43774642_43774659del	c.168+2_168+19del	p.Val23_Lys56delVal57fs*28 [11,20]
2 intron	g.43774637_43774656delinsTT	c.168+2_168+21delinsAA	Splice Site [11]
2 intron	g.43774332T>C	c.169-2A>G	Splice site [14]
3	g.43774306C>T	c.193G>A	p.Val65Ile [21]
3	g.43774297G>A	c.202C>T	p.Arg68ter [16,17,23,24]
3	g.43774287T>G	c.212A>C	p.Gln71Pro [24]
3	g.43774281dup	c.218dup	p.His75Ser_fs*2 [25]
3	g. 43774284_43774285del	c.218_219del	p.Leu73Pro_fs*3 [17,18,26]
5′ UTR_1	g.43776444_43776479ins(500_900)		[11,17,20,22,24,27,28]
5′ UTR_1	g.43776468_43776479del		[18]
5′ UTR_1	g.43776444_43776479insN(1500_1600)		[29]

## 2. Clinical Picture

EPM1 patients manifest photo-sensitive seizures, stimulus-sensitive myoclonus, tonic–clonic seizures, nocturnal myoclonic seizures, ataxia and dysarthria. The disease usually starts with nonvoluntary myoclonic jerks and/or tonic–clonic seizures that become clinically apparent between ages 6 and 15. The myoclonus is action-activated and stimulus-sensitive and occurs predominantly in the proximal muscles. Around half of the patients also present tonic–clonic seizures. The frequency of epileptic seizures tends to increase as the disease progresses [4]; however, the epileptic seizures usually respond well to antiseizure medication and may cease completely with appropriate treatment. The myoclonus, on the other hand, is less responsive to treatment and usually disabling. The clinical severity of the disease is quite heterogeneous and varies significantly between patients.

The EPM1 disease course is progressive, with some patients showing significant fluctuation for several years before losing their ability to walk [4]. The mental status of the patients is alert, but depression and mild decline in intellectual performance are seen as the disease progresses. Many patients also show comorbidities, with the common categories being behavioral disorders, endocrine disease, external injuries and mental, metabolic and nutritional diseases [30].

### Neurological Findings

The patients present a spike-and-wave-like graph on EEG [27,31,32], and cerebral atrophy, degenerative brain changes and loss of Purkinje cells are seen in MRI studies [33,34,35]. Further, the patients have decreased inhibitory control of the cerebral cortex by the cerebellum and the brainstem [36]. Recent studies with transcranial magnetic stimulation–electromyography (TMS-EMG) have revealed impairment in GABAergic inhibition in some EPM1 patients. Both impairments of short-interval intracortical inhibition (SICI) and long-interval intracortical inhibition (LICI) were seen in the study, and the impairment of SICI and LICI was directly proportional to the severity of the genetic defect [37].

## 3. Molecular Genetics of EPM1

EMP1 is inherited in an autosomal recessive manner and caused by biallelic mutations in the *CSTB* gene. *CSTB* is located on chromosome 21 at 21q22.3. The small gene consists of three exons that span 3 kb of genomic DNA (Figure 1) [16]. The gene encodes the Cystatin B protein, a cysteine protease inhibitor also known as Stefin B.

Various nonsense, missense and frameshift mutations have been identified in *CSTB* in EMP1 patients (Figure 1, Table 1). All known mutations are considered to result in a loss of function. The most common mutation, present in a homozygous form in the majority of the patients, is an unstable dodecamer repeat expansion of the 5′-CCCCGCCCCGCG-3′ sequence in the promoter region of *CSTB* [14,38] (Figure 1). The repeat expansion accounts for 90% of all EPM1 cases worldwide and 99% of Finnish EPM1 cases [4]. In healthy individuals, two or three copies of the repeat are present, whereas EPM1-associated alleles have been reported to contain at least 30 repeat copies, with up to 80 copies present in some patients. The expansion results in a significant reduction in the expression of the CSTB mRNA and protein in patients’ cells, and the loss of CSTB expression leads to the myoclonus and seizures in patients [20]. The length of the expansion mutation may correlate with the disease onset and the severity of the myoclonus and cortical neurophysiology [39]. There are few cases reported with individuals presenting alleles of 12–17 repeat copies; however, while these individuals do not manifest a severe disease, they have not been thoroughly clinically studied, so whether they are completely healthy is unknown [14].

## 4. The CSTB Protein

The CSTB protein consists of 98 amino acids and has a molecular weight of 11 kDa [40]. The monomeric protein structure contains one α-helix chain and a β-sheet of five β-strands; however, in cells, the protein commonly exists in an oligomeric form, and the oligomerization is sensitive to redox environment [41]. The tendency to oligomerize results in aggregate formation, and thus, the overexpression of the CSTB protein generates cytoplasmic aggregates [41]. CSTB functions as an inhibitor of the intracellular thiol proteases. The protein inhibits cathepsins L, H and B as well as papain. It is considered a key player in the regulation of cellular proteostasis, protecting against proteases leaking from lysosomes [19], and it is suggested that it also possesses a chaperone-like function [42].

### 4.1. CSTB Expression and Localization in the Brain

CSTB is widely expressed in different tissues and cell types but shows different expression levels and subcellular localization in different cell types [12,43]. In neural cells, CSTB is already expressed in embryonic and adult proliferative neural stem cells (NSCs). After the differentiation of NSCs into neurons or glial cells, the protein levels increase further [6,12,44].

The expression pattern of Cstb protein is widely studied in rodent models. The pattern shows distinct changes in the rat cerebellum during development. At day 4, several strong signals are detectable in inner granule layer cells. At day 7, the signal intensity increases in both external and internal granule layers, and from day 10 to day 17, as the expression intensity increases in the molecular layer, the molecular layer and the inner granule region are distinguishable. Finally at day 90, the protein is not detectable in the inner granule region but extends from the cell’s body at the molecular region border to the dendrites [44].

In situ hybridization and immunochemistry studies on adult rat brains show that the Cstb mRNA and protein are distinctly and highly expressed in the formation of hippocampal and reticular thalamic nucleus regions. The mRNA is also present in the amygdala, thalamus, hypothalamus and cortical areas in moderate levels [12,45]. In the adult rat cerebellum, the Cstb mRNA is detected only in Purkinje cells and Bergmann fibers and in dentate gyrus cortical neurons of the hippocampus [44]. In mice, however, the Cstb protein is widely expressed in the granule cell layer of the cerebellum [46]. Similarly to rats, in humans, the CSTB protein was not detectable in the cerebellum but was present in Purkinje cells and Bergmann glial fibers [44]. Cstb has also been detected in the synaptosomes in rat and mouse brain cortex, and this finding was verified in human cells in organoid cultures [47].

Both the nuclear and cytoplasmic localization of the Cstb protein has been detected in neurons; however, the function of the protein in the different compartments is still unclear. In neuroblastoma cell lines, the nucleus of proliferating cells shows a high expression of CSTB [43,48]. Similarly, in the cerebellum layer of a mouse brain, the localization is primarily nuclear [44,46]. After the differentiation of NSCs, the protein was present in the nucleus of both neurons and astrocytes, but in astrocytes, the protein was also found in the cytoplasm, whereas in neurons, cytoplasmic localization was not detected [6,12]. In astrocytes, the Cstb protein is further present in lysosomes, and mitochondrial localization of the Cstb protein has been reported in rat cerebellar granule cells [12,48].

### 4.2. CSTB Plays a Role in Brain Development

The varying distribution and localization of Cstb in neurons suggests that it can contribute to different cellular functions in the brain. In the rat cerebellum, the Cstb protein has been shown to interact with several cytoplasmic proteins, which are known to play roles in cellular growth, proliferation and differentiation [7]. Two hybrid studies have shown that Cstb interacts with brain β-spectrin, a cytoskeletal protein that aids in forming different complexes and membrane domains, as well as with NF-L, neurofilament light chain, which plays a key role in the cyto-dynamics and cytoarchitecture specification [7,44,49]. In addition, Cstb interacts with the cytoplasmic RACK-1 (receptor for activated C kinase 1) protein, which regulates the interaction between the cell membrane, cytoskeleton and the activated kinase [7,44,50]. RACK-1, β-spectrin and NF-L proteins co-localize in Purkinje cells and in Bergmann glia, where Cstb protein interacts with them, and together they guide development and differentiation [44].

**Figure 2:**
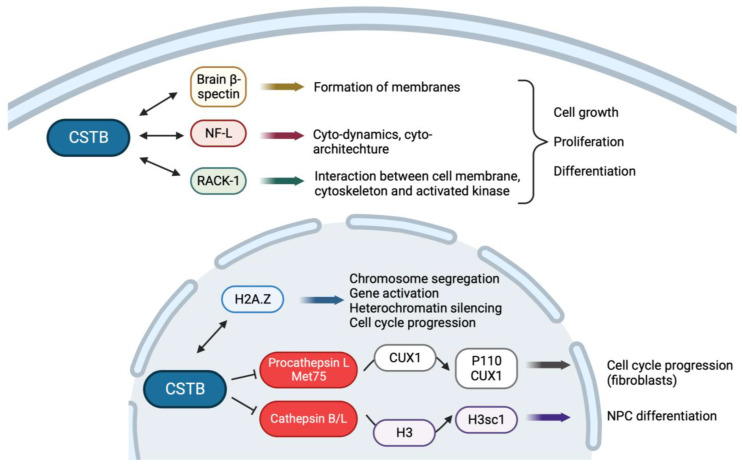
Interactions of CSTB protein in the cytoplasm and nucleus control cell proliferation and differentiation in a cell-type-specific manner. Created with Biorender.com (accessed on 7 December 2023).

The *Cstb* knockout mice provide a powerful tool for assessing the role of Cstb in the brain. In the *Cstb*-deficient mice, the loss of cerebellar granule cells and the loss of Purkinje cells were detected [34]. Similar findings can also be seen in autopsy samples from EPM1 patients [31,46,51], suggesting that Cstb is important for the development of the cerebellum. This may occur through its interaction with β-spectrin, NF-L and RACK-1 (Figure 2).

In the mouse model, Cstb downregulation results in reduced neuronal proliferation and number, reduced distribution of progenitors and their premature differentiation into neurons [10,52]. The number of neurons was reduced, and both the neuronal cell density as well as the total cell number in the cerebellar granule cell layer were significantly lower in the mutant mice than in healthy control mice. This leads to progressive atrophy as well as thinning of the cortex, and this likely underlies the cerebral ataxia. The degeneration of cortical neurons in the knockout mice takes place before any thalamic relay neuron loss [53,54,55]. The significant reduction in the neocortex thickness is further linked with the loss of GABAergic interneurons in older mice. The reduced GABAergic terminal number and decreased ligand binding to GABA receptors in the cerebellum were observed already in presymptomatic mice, and these events were also reported in an EPM1 patient [56].

Several of the molecular findings from the murine model were further confirmed in a human cell model. Di Matteo et al. generated human cerebral organoids (hCOs) from control and EPM1 patient cells [9]. In the human model, CSTB induced the recruitment of migrating interneurons in the organoids, and the level of the protein was critical in controlling progenitor cell proliferation. The overexpression of CSTB led to the expansion of progenitor cells, while a knock-down decreased the progenitor cell number. Interestingly, this control of cellular proliferation was cell non-autonomous. Similarly, reduced proliferation, reduced recruitment of interneurons and premature differentiation of progenitor cells were seen in the patient-derived hCOs with *CSTB* mutations [9]. These results from human cells show that the murine model faithfully replicates key findings from patient neurons and suggests that the function of CSTB is not only as a protease inhibitor but also as a key player in regulating cell proliferation, differentiation and neuronal migration.

### 4.3. CSTB Regulates Cell Cycle

The nuclear localization of CSTB has prompted studies on the nuclear functions of the protein. Ceru et al. [57] showed that the Cstb protein regulates cell cycle progression by interacting with histones and that it plays an important role in the regulation of cathepsin L proteolytic function in the nucleus. The effects of Cstb on the cell cycle seem to be tissue and cell-type specific. Embryonic fibroblast cells (mefs) from *Cstb* deficient mice enter the G1 cell cycle phase earlier than the control cells, while the overexpression of Cstb in glioblastoma cells led to the delayed progression of the cell cycle. These data suggest a critical role for Cstb in cell proliferation [57]. A co-immunoprecipitation assay in glioblastoma cells showed that Cstb interacts with histone H2A.Z in the nucleus (Figure 2). Histone H2A.Z plays a key role in chromosome segregation, gene activation, heterochromatin silencing and cell cycle progression [57,58,59].

Shorter procathepsin L isoforms, especially the procathepsin L Met 75 isoform, translocate to the nucleus, where procathepsin L Met75 cleave and process the CUX1 (Cut like homeobox 1) transcription factor into p110 CUX1, which accelerates entry into S Phase and promotes cell cycle progression [60,61,62]. The cathepsin L proteolytic activity is regulated by Cstb in the nucleus, and fluorescence resonance energy transfer (FRET) showed that Cstb interacts with procathepsin L Met75 isoform to regulate and limit its proteolytic function within the nucleus. Moreover, cells overexpressing Cstb have low p110 CUX1 levels due to the strong inhibition of cathepsin L function, leading to the low cleavage of CUX1 and, thus, low p110 levels [57] (Figure 2).

This negative effect of Cstb on cell cycle progression seen in fibroblasts and glioblastoma cell lines is opposite to what has been reported in neural progenitors. In human-derived cerebral organoids and in developing mice cortex, CSTB overexpression results in increased proliferation and affects specific cells in the S-phase, while the downregulation of CSTB showed an opposite effect. Further, cerebral organoids derived from EPM1 patients’ cells showed decreased cell proliferation and premature differentiation of neural cells [9]. These studies indicate that CSTB regulates cell proliferation in the developing cortex and that the effect on cell proliferation is cell-type specific.

A recent study suggests another nuclear function for the Cstb gene that may regulate the cell cycle changes in neural progenitors. Mouse neural progenitor cells (NPCs) undergo transient proteolytic cleavage of the N-terminal histone H3 tail upon differentiation. Cathepsins B and L, proteases associated with chromatin remodeling, are responsible for this cleavage at position T22, forming H3sc1. *Cstb* deficiency triggers premature cleavage in undifferentiated progenitor cells and sustained proteolysis in differentiating cells [63,64,65,66]. In *Cstb* deficient mice, the level of H3sc1 was, thus, high due to the high proteolytic activity of Cathepsins B and L, and this led to the early differentiation of the NPCs [66] (Figure 2).

These studies suggest that Cstb can regulate the cell cycle by regulating the proteolytic functions of cathepsins. Cathepsins, on the other hand, can either promote cell cycle progression or induce cell differentiation depending on the target of their proteolytic activity. This allows Cstb to regulate cell proliferation and differentiation in a cell-type-specific manner.

### 4.4. CSTB Regulates Mitochondria and Protects against Oxidative Stress

**Figure 3:**
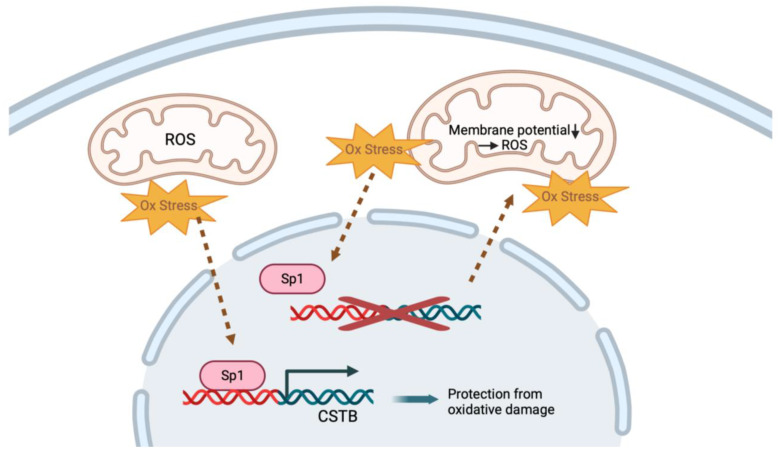
Cstb is involved in protection against oxidative stress. Created with Biorender.com (accessed on 7 December 2023).

The Cstb protein has also been reported to protect neurons from oxidative stress and to regulate mitochondrial function. In healthy cells under oxidative stress, the sp1 transcription factor binds to the promoter region of *Cstb* and promotes its expression, which is essential in protecting against oxidative stress and preventing cellular damage and cell death [67,68] (Figure 3). In *Cstb*-mutant mice, the loss of the Cstb protein leads to increased oxidative stress, which induces the destabilization of the mitochondrial membrane potential and further aggravates mitochondrial superoxide generation [43]. Moreover, antioxidant enzymes, like superoxide dismutase (SOD), glutathione (GSH) and catalase, were significantly reduced in the cerebellum of the mutant mice, which resulted in the accumulation of free radicals that led to cellular damage and oxidative stress-induced cell death [68]. Also, differentiating mutant mouse NPCs showed increased levels of mitochondrial reactive oxygen species (ROS) and decreased antioxidant levels.

Mitochondrial dysfunction seems to be critical for EPM1 pathogenesis. The downregulation of Cystatin B in mice leads to transcriptional changes in nuclear-encoded mitochondrial genes, which causes mitochondrial respiration dysregulation in the mutant NPCs, together with the premature differentiation of the cells [66]. Further, a proteomic analysis of cerebellar synaptosomes from presymptomatic *Cstb*-deficient mice suggests that mitochondrial dysfunction is an early event in EPM1 pathogenesis [8]. In the study, one-third of all differentially expressed proteins in synaptosomes were identified as mitochondrial, and the fold changes were higher for mitochondrial proteins than for the other proteins. Respirometry analysis revealed that the mitochondrial dysfunction in *Cstb*-deficient mice is progressive and coincides with the onset of neurodegeneration and myoclonus [69], further suggesting a critical role for mitochondria in EPM1.

### 4.5. CSTB in Neuroinflammation

Cstb expression is upregulated upon cellular stress and macrophage/microglial activation [43]. Microglia are the innate immune cells of the central nervous system. Among their key roles are to phagocytize foreign invaders, remove cellular debris, assist in wound repair and initiate immune responses. In addition, they are also involved in maintaining normal cellular homeostasis, neuronal survival and synaptic functions [70]. Microglial cells are affected in EPM1 patients [55], and early microglial activation is already seen in 2-week-old *Cstb*-knockout mice, together with the extensive activation of astroglia and preceding selective neuronal loss [10,55]. Both pro-inflammatory M1 and anti-inflammatory M2 microglia are upregulated in the *Cstb*-deficient mice, and high expressions of both pro-inflammatory marker inducible nitric oxide synthase (iNOS) and anti-inflammatory marker arginase 1 (ARG1) have been observed in the mouse cortex [10]. As the disease progresses, microglia change their morphology from phagocytic round cells at early disease stages to cells with thickened and branched processes at the later stages [55]. The glial cell activation in *Cstb*-knockout mice leads to a high mRNA expression of the C1q B-chain of the complement (*C1qB*), β2-microglobulin, glial fibrillary acidic protein (*Gfap*), apolipoprotein D and fibronectin. These genes are typically predominantly expressed in activated microglia and enhance the microglial clearance of apoptotic cells while suppressing proinflammatory cytokines. They also increase the activity of reactive astrocytes [71,72,73,74]. The activated microglia in the mutant mice show elevated chemokine release and chemotaxis. Further, the expression of the p-p38 MAPK was higher in mutant mouse cortex in comparison to healthy mice [10]. The p38 MAPK cascade regulates a variety of cellular responses to stress and inflammation. In its nonphosphorylated form, the cascade is fairly inactive but becomes rapidly activated upon phosphorylation [75].

*Cstb*-deficient mice were shown to be more sensitive to lipopolysaccharide (LPS)-induced sepsis due to increased caspase-11 expression [43]. They also secreted higher levels of pro-inflammatory cytokines. In macrophages, LPS stimulation leads to the translocation of Cstb into mitochondria, and without Cstb, oxidative stress is increased. Further, increased caspase-11 expression results in the activation of the non-canonical inflammasome in macrophages. A similar pathway is likely also present in microglia, and oxidative stress may play an important role in non-canonical inflammasome activation and neuronal cell death in EPM1 [43].

### 4.6. CSTB in GABAergic Neurons

The wide expression of CSTB in different neuronal cells suggests that several different neuronal types may be affected in EPM1 patients; however, the loss of inhibitory control points to GABAergic cells as a putative target for treatment strategies. GABAergic neurons develop early during embryonic development in the cortical anlage [76]. They produce the gamma-aminobutyric acid (GABA) neurotransmitter, which plays a key role in regulating neuronal proliferation, migration, differentiation and preliminary circuit-building. In the mature brain, GABA is the main inhibitory neurotransmitter of the mammalian central nervous system [77]. GABAergic neurons exert a presynaptic action via GABA receptors and inhibit dopaminergic neurons [78]. GABA has been shown to be involved in motor control, vision and cortical functions, and GABA dysfunction is associated with epilepsy, stiff-person syndrome and schizophrenia [19]. Recent studies have shown that EPM1 patients and the *Cstb* knockout mice have a dramatic reduction in the thickness of the cortex and a clear loss of GABAergic neurons. Electrophysiological studies showed that this results in altered GABAergic signaling with a subsequent loss of GABA inhibition, suggesting a mechanism for the latent hyperexcitability resulting in myoclonus and seizures in the patients [8,34,56,79]. However, while the expression of the sodium- and chloride-dependent GABA transporter 1 (GAT-1) was decreased in the synaptosomes of *Cstb*-deficient mice, no functional differences in GABA responses were detected in electrophysiological experiments, in comparison to healthy littermates [8]. Despite this, the current hypothesis is that the reduced GABAergic cells and alterations in GABAergic signaling result in decreased inhibition in the cerebellum, which leads to the hyperexcitable phenotype and the seizures [34,56]. Overall, while Cstb clearly plays an important role in GABAergic neurons, the full spectrum of the cellular functions it is involved in is not yet understood, and the role that GABAergic neurons have in EPM1 pathogenesis still needs further studies.

### 4.7. CSTB in Synaptosomes

In *Cstb* knockout mice, the altered expression of genes involved in synaptogenesis was observed [34]. Further, a proteomics study on the cerebellum showed the altered expression of mitochondrial, ribosomal and intracellular transport proteins in synaptosomes. The altered proteins play key roles, for example, in mitochondrial function, synaptic signaling, mRNA translation and cytoplasmic signaling. These findings suggest that aberrant synaptic signaling in *Cstb*-deficient mice could arise from altered bioenergetics in the synaptosomes, which is due to early mitochondrial dysfunction [8,69].

Further, Cstb was shown to be locally synthesized in synaptosomes and secreted from them in a depolarization-controlled manner [47]. The secreted Cstb seems to be involved in the systematic recruitment and migration of interneurons. Kinesin-like protein Kif1a is a neuron-specific member of the kinesin-3 motor protein family that uses ATP hydrolysis to create mechanical force and “walks” along microtubule filaments to transfer cargo from the neuronal cell body to the neurites [80]. Cstb was shown to interact with Kif1a, and the secretion of Cstb is dependent on Kif1a. The secreted Cstb guides the recruitment and migration of interneurons, and in Cstb mutant mice, interneuron migration is dysregulated when compared to healthy controls [9]. The strong presence of Cstb in synaptosomes and the depolarization-controlled secretion suggest that Cstb may be involved in controlling brain plasticity.

## 5. Conclusions

EPM1 is a rare genetic disorder that affects the central nervous system, resulting in seizures, myoclonic jerks and progressive neurological deterioration. EPM1 is caused by mutations in the gene encoding CSTB, which normally regulates proteases in the brain. The disease typically first presents in childhood or adolescence and gradually worsens over time, leading to cognitive decline, ataxia and muscle weakness. Genetic counseling and testing are recommended for affected individuals and their family members.

The various cellular localizations, i.e nuclear, cytoplasmic, lysosomal and mitochondrial of the Cstb protein suggest that the protein may also have various functional roles. These may occur through interaction with other molecules and induce or inhibit downstream signaling pathways or through playing a role in regulating gene expression. In addition, while fairly ubiquitously expressed, the different localizations of Cstb in different cell types suggest that some of the functions may be cell-type specific. Cstb has several neuronal functions. It functions as a cysteine protease inhibitor, regulates cell proliferation through cell cycle regulation and regulates mitochondrial function in neurons. Mitochondrial function plays a crucial role in maintaining neuronal function, plasticity and apoptosis.

To date, more than 100 patients with *CSTB* mutations have been reported. There is no cure for EPM1, and treatment is largely supportive and aimed at controlling seizures and other symptoms. Medication, physical and speech therapy and nutritional support can help manage symptoms and prevent complications. However, current therapies only control the symptoms or aim to avoid future complications and do not improve cognitive function. Also, current therapies do not allow the patients to perform their everyday activities normally. In the majority of the patients, the epileptic seizures can be controlled well; however, the myoclonus is less responsive to existing pharmacological options and significantly reduces the quality of the patient’s life; thus, additional research and novel treatment options are needed.
